# Supplementary material for: Feeding Drosophila highly radioresistant fungi improves survival and gut morphology following acute gamma radiation exposure
Source: bioRxiv. 2025 Jul 11:2025.07.10.664168. Preprint. [Version 1] doi: 10.1101/2025.07.10.664168 (PMC12265717; doi:10.1101/2025.07.10.664168)
Supplement: Supplement 3 [file media-3.pdf]

Supplementary Table 1. Influence of Dietary Fungi on Radiation Survival

Group	Dose	Diet	Mean	S.E.	Log Rank Bonferroni P-value			Gehan-Breslow-Wilcoxon P-value		
					P (vs Control)	P (vs Aureo+Mel)	P (vs Aureo-Mel)	P (vs Control)	P (vs Aureo+Mel)	P (vs Aureo-Mel)
Female	1000 Gy	Control	15.85	0.5	-	1	1	-	0.461	1
Female	1000 Gy	Aureobasidium(+Mel	14.65	0.74	1	-	0.8718	0.461	-	0.6429
Female	1000 Gy	Aureobasidium(-Mel)	15.55	0.73	1	0.8719	-	1	0.6429	-
Male	1000 Gy	Control	12.45	0.34	-	0.0086	0.0142	-	0.0331	0.0297
Male	1000 Gy	Aureobasidium(+Mel	13.9	0.48	0.0086	-	0.8374	0.0331	-	1
Male	1000 Gy	Aureobasidium(-Mel)	13.65	0.41	0.0142	0.8374	-	0.0297	1	-
**Group**	**Dose**	**Diet**	**Mean**	**S.E.**	**Log Rank Bonferroni P-value**			**Gehan-Breslow-Wilcoxon P-value**		
					**P (vs Control)**	**P (vs Rhodo+Ɣ)**	**P (vs Rhodo-Ɣ)**	**P (vs Control)**	**P (vs Rhodo+Ɣ)**	**P (vs Rhodo-Ɣ)**
Female	700 Gy	Control	23.3	0.75	-	0.0001	0.0062	-	0.0002	0.0116
Female	700 Gy	Rhodotorula(+Ɣ)	17.45	1.04	0.0001	-	0.2444	0.0002	-	0.0783
Female	700 Gy	Rhodotorula(-Ɣ)	20.6	0.54	0.0062	0.2444	-	0.0116	0.0783	-
Male	700 Gy	Control	17.4	0.57	-	0.0143	1	-	0.0041	1
Male	700 Gy	Rhodotorula(+Ɣ)	14.45	0.84	0.0143	-	0.0664	0.0041	-	0.0407
Male	700 Gy	Rhodotorula(-Ɣ)	16.85	0.63	1	0.0664	-	1	0.0407	-

* for average of n=20 triplicate
